# The Role of ^99m^Tc-Annexin V Apoptosis Scintigraphy in Visualizing Early Stage Glucocorticoid-Induced Femoral Head Osteonecrosis in the Rabbit

**DOI:** 10.1155/2016/7067259

**Published:** 2016-02-16

**Authors:** Xiaolong Wang, Yu Liu, Xuemei Wang, Rui Liu, Jianbo Li, Guoliang Zhang, Qiang Li, Lei Wang, Zhigang Bai, Jianmin Zhao

**Affiliations:** ^1^Department of Surgery, Affiliated Hospital of Inner Mongolia Medical University, No. 1 Tongdao North Street, Hohhot, Inner Mongolia 010050, China; ^2^Department of Nuclear Medicine, Affiliated Hospital of Inner Mongolia Medical University, No. 1 Tongdao North Street, Hohhot, Inner Mongolia 010050, China; ^3^Fuwai Hospital, Beijing, China

## Abstract

*Objective*. To validate the ability of ^99m^Tc-Annexin V to visualize early stage of glucocorticoid-induced femoral head necrosis by comparing with ^99m^Tc-MDP bone scanning.* Methods*. Femoral head necrosis was induced in adult New Zealand white rabbits by intramuscular injection of methylprednisolone. ^99m^Tc-Annexin scintigraphy and ^99m^Tc-MDP scans were performed before and 5, 6, and 8 weeks after methylprednisolone administration. Rabbits were sacrificed at various time points and conducted for TUNEL and H&E staining.* Results*. All methylprednisolone treated animals developed femoral head necrosis; at 8 weeks postinjection, destruction of bone structure was evident in H&E staining, and apoptosis was confirmed by the TUNEL assay. This was matched by ^99m^Tc-Annexin V images, which showed a significant increase in signal over baseline. Serial ^99m^Tc-Annexin V scans revealed that increased ^99m^Tc-Annexin V uptake could be observed in 5 weeks. In contrast, there was no effect on ^99m^Tc-MDP signal until 8 weeks. The TUNEL assay revealed that bone cell apoptosis occurred at 5 weeks.* Conclusion*. ^99m^Tc-Annexin V is superior to ^99m^Tc-MDP for the early detection of glucocorticoid-induced femoral head necrosis in the rabbit and may be a better strategy for the early detection of glucocorticoid-induced femoral head necrosis in patients.

## 1. Introduction

Glucocorticoid-induced necrosis of the femoral head (also known as glucocorticoid-induced avascular necrosis or aseptic necrosis of the femoral head) was initially reported in the 1950s [1]. In China, it is estimated that there are approximately 5 to 7.5 million patients suffering from femoral head necrosis, with 75,000 to 150,000 new cases each year [2]. Glucocorticoid is the primary cause of nontrauma femoral head necrosis, a condition which seriously reduces the patient's quality of life and ability to work, and eventually requires artificial joint replacement in the majority of cases. If femoral head necrosis can be detected at a very early stage, it may be possible to prevent the collapse of the femoral head, preserving joint function, delaying progression of the disease, leading to an overall reduction in morbidity [3–5].

In previous decades, the pathogenesis of glucocorticoid-induced osteonecrosis has been ascribed to increasing intraosseous pressure and decreased blood perfusion in the bone, fat embolism, or a hypercoagulable state [5]. Recently, it has been demonstrated that glucocorticoid-induced bone of femoral head necrosis is closely associated with bone cell apoptosis, and, using a rabbit model, Hwang et al. demonstrated the early appearance of apoptosis after glucocorticoid treatment [6]. Youm et al. found that high dose glucocorticoid significantly increased apoptosis in osteoblast and osteoclast cells in the femoral head, with subsequent trabecular bone fracture and femoral head collapse; further, apoptosis of osteoblasts reduced bone reconstruction and repair [7, 8], without affecting the vascular supply to the femoral head [8]. In transplant patients, Rojas et al. demonstrated osteoblastic cell apoptosis after high dose glucocorticoid [9], further supporting a role for programmed cell death in glucocorticoid-induced femoral head necrosis in patients and animals.

^99m^Tc-Annexin V is a radionuclide-labeled molecular probe for detecting apoptosis that has been validated in animal experiments and preliminary clinical use [10, 11]. ^99m^Tc-Annexin V imaging successfully detected apoptotic cells in acute myocardial infarction, myocarditis, cardiac transplant rejection, unstable atherosclerotic plaque, and cancer after effective chemotherapy [11]. Recently, ^99m^Tc-Annexin V has been used to monitor the therapeutic effect of antiapoptosis drugs in heart failure patients [12–14]. We hypothesized that ^99m^Tc-Annexin V scintigraphy would successfully image apoptosis in the femoral head and so enable visualization of early stage disease.

In this study, using a rabbit model, glucocorticoid-induced necrosis of the femoral head was imaged with both ^99m^Tc-Annexin V and ^99m^Tc-MDP bone scan. Comparison of the two imaging agents suggests that ^99m^Tc-Annexin V may be superior.

## 2. Materials and Methods

### 2.1. Animals

Adult New Zealand white rabbits were purchased from Di Dunlop Biological Resources Development Co. (Xi'an, China). Rabbits were maintained and used according to the guidelines of Inner Mongolian Medical University Animal Care and Use Committee. The animal study protocols were approved by the committee. Rabbits were housed singly and supplied with standard diet.

### 2.2. Generation of a Rabbit Model of Glucocorticoid-Induced Femoral Head Necrosis

Experiments were performed on 20 rabbits (body weight 2.5 ± 0.3 kg, 10 male, 10 female). Animals were acclimated in the vivarium for one week and injected intramuscularly with methylprednisolone (7.5 mg/kg), twice weekly for 8 weeks to generate femoral head necrosis. Six control rabbits received saline by the same route and schedule.

### 2.3. ^99m^Tc-Annexin V Scintigraphy and ^99m^Tc-MDP Bone Scans

Annexin V was labeled in-house as previously described [10–14], to a radiochemical purity of 90%. 0.5 mCi (18.5 MBq) was injected via the ear vein and imaging was performed 1 hour later. Bone scintigraphy was performed 48 hours prior to ^99m^Tc-Annexin V imaging. ^99m^Tc-MDP (0.5 mCi [18.5 MBq], radiochemical purity 95%) was injected via the ear vein and imaged 2 hours subsequently.

Imaging was performed with a clinical dual-head detector SPECT/CT (Millennium VG, Hawkeye; GE Healthcare) equipped with low-energy and high-resolution collimators (peak energy 140 keV, window width 20%). Animals were anesthetized by inhalation of isoflurane (1.5–2%)-air mixture for a 6 min planar scan. SPECT data was acquired into a 128 × 128 matrix.

The images were processed using a postprocessing system workstation. Tracer uptake was expressed as the ratio of signal in limb joints to mediastinum (T/N). And images were read by two nuclear medicine physicians who were blinded to the pathologic findings. In case of disagreement, a third nuclear medicine physician made a diagnostic conclusion.

### 2.4. Preparation of Femoral Head Paraffin Sections for Histological Assay

Rabbits were sacrificed either by intravenous injection of sodium pentobarbital or air embolism. The femoral heads were resected and stored in 10% formalin solution. Femoral heads were decalcified by immersing in Surgipath II decalcification solution (10% formaldehyde, 8% formic acid, and 1% methanol; Surgipath, Richmond, USA) for approximately 1 week and subsequently embedded in paraffin. Blocks were sectioned (4 *μ*m slices) every 1 mm throughout the bone.

### 2.5. TUNEL Assay

Terminal deoxynucleotidyl transferase- (TDT-) mediated dUTP nick end labeling (TUNEL) was performed according to the kit manufacturer's instructions (Thermo Scientific, USA) for tissue sections. Slices were dewaxed with freshly prepared 3% H_2_O_2_ solution at room temperature for 10 min and treated with proteinase K at 37°C for 10 min. Tissue was covered with 20 *μ*L labeling buffer containing TDT (1 *μ*L) and DIG-dUTP (1 *μ*L) and incubated for 2 h at 37°C in 100% humidity. Biotinylated antidigoxigenin antibody was applied (50 *μ*L per specimen, 37°C for 30 min) followed by SABC (10 *μ*L, 37°C, 30 min) and finally stained with hematoxylin for 2 s. TUNEL-positive cells were identified through the nucleus, which was either stained tan or brown. Five fields were randomly selected and the osteoblast apoptosis index was calculated as the ratio of apoptotic to total cells.

### 2.6. Statistical Analysis

SPSS19.0 software (IBM, USA) was used for statistical analysis. Data was expressed as mean ± standard deviation. Student's *t*-test was used to compare differences between groups, and a *P* value less than 0.05 was considered statistically significant.

## 3. Results

**Figure 1:**
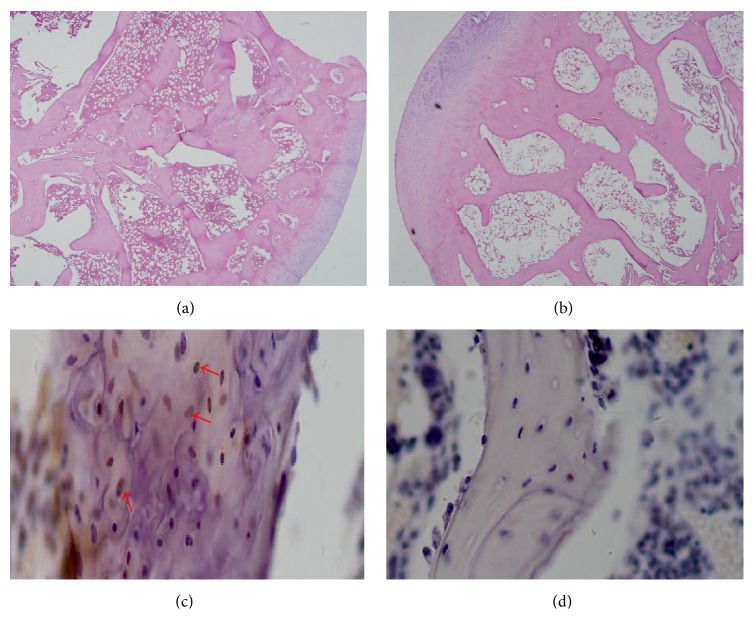
Histological images demonstrating methylprednisolone-induced femoral head necrosis. (a) Representative H&E stained section of a femoral head, 8 weeks after methylprednisolone administration, showing destruction of the bone structure (2 rabbits were examined). (b) Representative H&E stained section of a femoral head section from a control rabbit treated with saline (2 rabbits were examined). (c) TUNEL assay of a femoral head, 8 weeks after methylprednisolone administration. Apoptotic cells (indicated by arrows) are stained brown. (d) TUNEL assay of a femoral head from a control rabbit treated with saline.

Five weeks following methylprednisolone injection, rabbits displayed decreased activity, malaise, a decrease in lower limb body support, and reduced food intake. All animals developed femur head necrosis as confirmed by H&E staining and TUNEL at 8 weeks (Figure 1).

**Figure 2:**
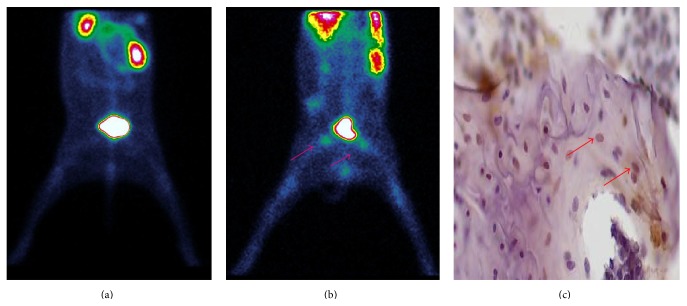
Representative ^99m^Tc-Annexin V images of methylprednisolone-induced femoral head necrosis. (a) ^99m^Tc-Annexin V image of a saline-treated rabbit as control. (b) ^99m^Tc-Annexin V image of a rabbit after 8 weeks of prednisolone treatment. Radioactivity accumulates in the femoral head regions as indicated by the arrows. (c) TUNEL assay of a femoral head section, 8 weeks after prednisolone administration, with apoptotic cells stained brown, as indicated by the arrow.

Planar scintigraphy performed 1 day before and 8 weeks after methylprednisolone administration in 6 rabbits showed that ^99m^Tc-Annexin V accumulation in the femoral heads had significantly increased. (Representative ^99m^Tc-Annexin V images are presented in Figures 2(a) and 2(b).) Subsequent histology indicated the presence of elevated levels of apoptosis (Figure 2(c)).

**Figure 3:**
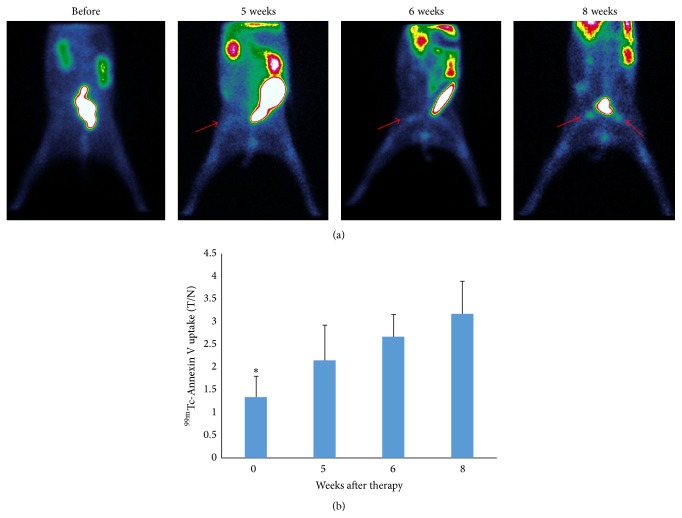
Serial ^99m^Tc-Annexin V scans in methylprednisolone treated rabbits. (a) Scans were performed before therapy and 5–8 weeks after initiation by methylprednisolone. Increasing ^99m^Tc-Annexin V uptake is observed as early as approximately 5 weeks after the start of prednisolone treatment. (b) ^99m^Tc-Annexin V uptake, expressed as T/N, significantly increased with time.

To observe the disease development process, we performed serial ^99m^Tc-Annexin V scans in 6 rabbits (3 male, 3 female). Scans were collected before treatment and 5–8 weeks after the initiation of methylprednisolone treatment. As shown in Figure 3(a), increased ^99m^Tc-Annexin V uptake was observed as early as approximately 5 weeks after commencing methylprednisolone. ^99m^Tc-Annexin V uptake, expressed as T/N, was significantly increased over baseline at all observed time points (Figure 3(b)).

**Figure 4:**
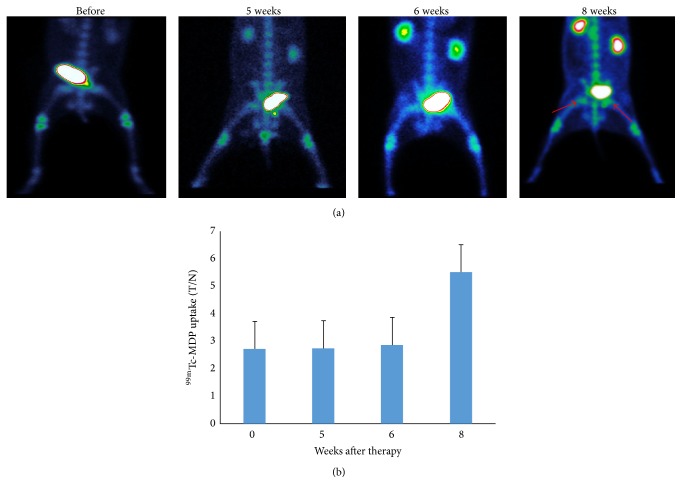
Serial ^99m^Tc-MDP bone scans in methylprednisolone treated rabbits. Animals from Figure 3 were scanned with ^99m^Tc-MDP, two days prior to ^99m^Tc-Annexin V imaging. (a) Abnormal ^99m^Tc-MDP is found by 8 weeks, as indicated by the arrow. (b) A significant increase in ^99m^Tc-MDP is observed only at 8 weeks after methylprednisolone exposure, ^*∗*^ *P* < 0.01 at 8 weeks versus the rest of the time points. There were no significant differences in ^99m^Tc-MDP uptake between pretherapy (week 0) and 5 or 6 weeks.

^99m^Tc-MDP bone scans were performed in the same group of animals two days prior to ^99m^Tc-Annexin V imaging. Abnormal femoral head uptake of ^99m^Tc-MDP was not observed until 8 weeks, when it was significantly higher than pretreatment (*P* < 0.01) (Figure 4).

**Figure 5:**
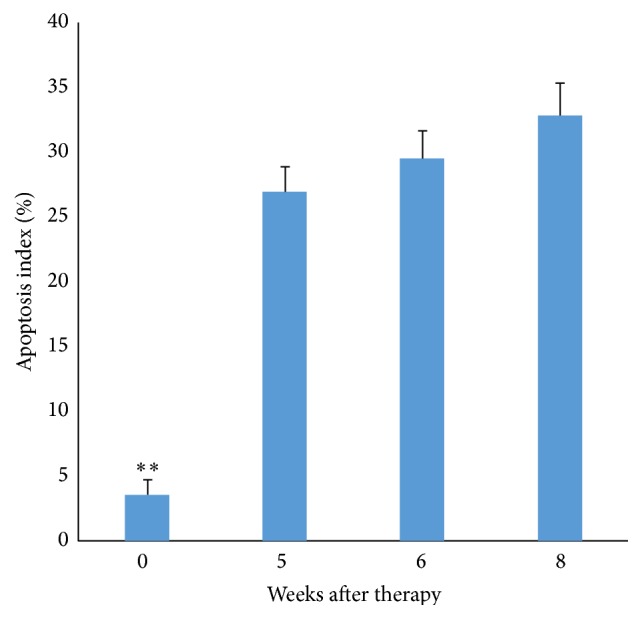
Apoptotic frequency as a function of time after treatment by TUNEL assay. The frequency of apoptosis as assessed by TUNEL in rabbits sacrificed at 5, 6, and 8 weeks after methylprednisolone treatment. Control animals were treated with saline. Each treated point represents 4 femoral heads and 8 in the control group, ^*∗∗*^ *P* < 0.001.

In a separate group, 6 rabbits were treated with methylprednisolone and sacrificed at 5, 6, and 8 weeks after treatment (two animals per time point and four control animals). The TUNEL assay revealed a significantly higher apoptotic index in the treated femoral heads at each endpoint (*P* < 0.05) relative to the controls (Figure 5).

## 4. Discussion

Animal models of glucocorticoid-induced femoral head osteonecrosis mimic human disease and are thus critically important for in-depth studies of both pathogenesis and therapy. Importantly, we have successfully established that, in methylprednisolone-induced femoral head osteonecrosis in rabbits, the pathogenesis was closely related to bone cell apoptosis (Figure 1). We have also used this model to validate that ^99m^Tc-Annexin V apoptosis imaging is superior to ^99m^Tc-MDP for the early detection of the disease (Figures 2–4).

Glucocorticoid treatment is the leading cause of nontraumatic femoral head necrosis [15]. However, in the asymptomatic early stage, the disease is difficult to diagnose with current imaging modalities. In 1971, Harrington et al. [16] were the first to describe bone avascular necrosis in renal transplant patients treated with glucocorticoids. Subsequently, the key role of apoptosis in the pathogenesis of glucocorticoid-induced necrosis was established [7]. Accordingly, in this study we hypothesized that apoptosis imaging may visualize early pathological changes in the femoral head. ^99m^Tc-Annexin V successfully detected the onset of femoral head necrosis after 5 weeks of continuous glucocorticoid administration (Figure 3) and was much more sensitive than a ^99m^Tc-MDP bone scan (Figure 4), which is currently considered the gold standard for the clinical diagnosis of femoral head necrosis. The imaging results were consistent with the TUNEL assay which demonstrated the presence of apoptosis 5 weeks after methylprednisolone administration.

Annexin V specifically binds to phosphatidylserine on the surface of early apoptotic cells and is widely used to detect apoptosis [17]. Radionuclide-labeled Annexin V has been used for the in vivo detection of apoptosis [18–20] and has been the subject of clinical trials in the US and Europe designed to assess chemotherapy of small-cell lung cancer [21]. To the best of our knowledge, ^99m^Tc-Annexin V has not yet been used to detect very early stage glucocorticoid-induced femoral head osteonecrosis.

^99m^Tc-Annexin V apoptosis imaging was found to be superior to ^99m^Tc-MDP bone scintigraphy for the early detection of glucocorticoid-induced femoral head osteonecrosis (Figures 3 and 4) and may be used to observe the therapeutic effect of novel treatment strategies. We are in the process of using ^99m^Tc-Annexin V SPECT to study and evaluate patients who have received high dose glucocorticoid for various reasons.

## 5. Conclusions

^99m^Tc-Annexin V apoptosis imaging is superior to ^99m^Tc-MDP bone imaging for the early detection of glucocorticoid-induced femoral head necrosis in the rabbit and may be a better strategy for detecting early stage femoral head necrosis induced by glucocorticoid in patients.
